# Culture-based viability PCR: strategies to harness sensitivity and minimize false positives

**DOI:** 10.1017/ash.2025.10071

**Published:** 2025-07-18

**Authors:** Bobby G. Warren, Aaron Barrett, Guerbine Fils-Aime, Amanda M. Graves, Deverick J. Anderson

**Affiliations:** 1 Duke Center for Antimicrobial Stewardship and Infection Prevention, Durham, NC, USA; 2 Disinfection, Resistance and Transmission Epidemiology (DiRTE) Laboratory, Durham, NC, USA; 3 Division of Infectious Diseases, Duke University Medical Center, Durham, NC, USA

## Introduction

A total of 700,000 healthcare-associated infections occur annually in the United States. The healthcare environment plays a key role in the spread of these infections, as pathogens survive on surfaces and contribute to transmission.^1,2^ As a result, detection and mitigation of pathogens in the healthcare environment is crucial for effective infection prevention.

Culture methods are the gold standard for detecting pathogens in healthcare settings. While these methods can confirm viable organisms, they have a high detection threshold, are slow, and require specialized personnel. Quantitative Polymerase Chain Reaction (qPCR) offers a faster alternative and is generally more sensitive then direct plating methods by detecting small amounts of DNA. However, it cannot distinguish between live and dead cells, as it detects genetic material that may persist after cell death.

Given the limitations of culture and qPCR, we explored the concept of culture-based viability PCR, a method that involves running uses species-specific qPCR before and after incubation in growth media to assess whether detected organisms can proliferate. This approach combines qPCR’s sensitivity with viability assessment, improving how we evaluate environmental contamination risks in healthcare settings.

## Methods

We completed a prospective microbiological analysis of patient bed footboard samples at Duke University Hospital in Durham, North Carolina. Eligible patient rooms included single occupant rooms housing patients with an active infection who were also on contact precautions. Target species included *E. coli* (EC), *Staphylococcus aureus* (SA), and *Clostridioides difficile* (CD).

Footboard samples were obtained via foam sponges premoistened in neutralizing buffer and processed via the stomacher method resulting in a 5 mL homogenate.^3^ Sponge homogenates were split into three paths: 1) T_0_: 500uL was added to 4.5 ml of trypticase soy broth (TSB); 500uL of the resulting mixture underwent DNA extraction and qPCR with species-specific primers,^4–6^ 2) T_1_: 500uL was added to 4.5 mL of TSB, and 3) Growth negative control (GNC): 500uL was added to 4.5 mL of 8.25% sodium hypochlorite, left at room temperature for 10 minutes, centrifuged for 15 minutes at 3 100 RPM, then decanted and added to 5 mL of TSB after 2 PBS washes.

T_1_ and GNC samples were then incubated at species specific conditions (24 hours at 37°C aerobically for *EC* and *SA*, and 48 hours anaerobically for *CD*). After incubation, 500uL from T_1_ and GNC samples underwent DNA extraction and qPCR. Additionally, 200uL of samples from all three paths were also cultured on TSA agar in parallel.

A sample was considered viable for each species if 1) it was detected at T_0,_ and the CT decreased by at least 1.0 at T_1_ compared to GNC or 2) it was undetected at T_0_, detected at T_1_, and undetected for GNC, or 3) grew on standard culture agar. All qPCR assays were performed using SYBR Green following manufacturer’s guidelines and in triplicate after DNA extraction, and results were averaged.^7^ The *Z* score proportionality test was used to compare the proportion of samples considered viable. *P* < .05 was considered significant, all statistical tests were 2-tailed, and all testing was completed using R software (R Foundation for Statistical Computing, Vienna, Austria).

## Results

We enrolled 26 patient rooms between March and April of 2024. Patients in these rooms had a median age of 59 Interquartile Range (IQR: 47 – 68) and a median length of stay of 25 days (IQR:12 – 66); 15 (54%) were male, 26 (100%) had an active infection and of those, and 12 (46%) had an active infection with a study pathogen.

A total of 468 samples from the 26 patient rooms were analyzed, including 156 for each species (26 rooms × 6 samples in each room for each organism). Of the 26 original samples evaluated by qPCR, 24 (92%), 11 (42%), and 2 (8%), had detectable levels of *EC*, *SA,* and *CD* via qPCR at T_0_ or T_1_, respectively, and could be assessed for viability. Of those, 3 (13%), 8 (73%), and 0 (0%) contained viable cells of *EC*, *SA* and, *CD* via qPCR, respectively, compared to 0 (0%), 0 (0%), and 0 (0%) via culture, respectively (*P* < .01). Notably, 5 (19%) of *SA* samples were detectable using culture-based methods at T_1,_ indicating broth enrichment enhanced culture sensitivity; however, all were determined viable via qPCR as well (Table 1). As expected, GNC sample results mirrored T_0_ samples, so these data are not shown.

**Table 1.** Detection proportions and viability results by species, detection method, and study time point

	T_0_	T_1_	T_1_
	Detected via qPCR	Detected via qPCR	Detected via qPCR and Viable
Overall (N = 78)	33 (42)	37 (47)	11 (30)
*E. coli* (N = 26)	24 (92)	24 (92)	3 (13)
*S. aureus* (N = 26)	7 (27)	11 (42)	8 (73)
*C. difficile* (N = 26)	2 (8)	2 (8)	0 (0)
	Detected via Culture		Detected via Culture and Viable*
Overall (N = 78)	0 (0)		5 (6)
*E. coli* (N = 26)	0 (0)		0 (0)
*S. aureus* (N = 26)	0 (0)		5 (19)
*C. difficile* (N = 26)	0 (0)		0 (0)

* All specimens deemed viable by standard culture methods after incubation were also identified by qPCR as viable.

## Discussion

Culture-based viability PCR outperformed traditional culture methods in detecting viable pathogens with improved specificity compared to qPCR highlighting its potential to better assess pathogen viability compared to standard qPCR alone and as a tool for assessing environmental contamination. The concept of culture-based viability PCR has been studied previously on *C. difficile* and *B. anthracis* spores but, to our knowledge, has not been deployed on vegetative cells, other species, or on real-world environmental samples.^8–10^ Therefore, this strategy is novel and could offer a more efficient and practical solution for routine environmental monitoring.

Our study had limitations. First, our sample size was relatively small, only one surface type was sampled, and the study was conducted at a single healthcare center, limiting generalizability. Next, the targeted species are all known to frequently contaminate and survive on surfaces for a relatively long period of time compared to other clinically relevant pathogens, which could have inflated the efficacy of this method. Future studies involving a broader range of pathogens and clinical settings are needed to validate these findings.

In conclusion, culture-based viability PCR should be considered as a tool for assessing the healthcare environment for clinically relevant bacteria.
